# Resting-State Neuronal Oscillatory Correlates of Working Memory Performance

**DOI:** 10.1371/journal.pone.0066820

**Published:** 2013-06-25

**Authors:** David Heister, Mithun Diwakar, Sharon Nichols, Ashley Robb, Anne Marie Angeles, Omer Tal, Deborah L. Harrington, Tao Song, Roland R. Lee, Mingxiong Huang

**Affiliations:** 1 Department of Radiology, University of California San Diego, San Diego, California, United States of America; 2 Neurosciences, University of California San Diego, San Diego, California, United States of America; 3 Radiology and Research Services, Veterans Affairs San Diego Healthcare System, San Diego, California, United States of America; University Medical Center Groningen UMCG, The Netherlands

## Abstract

**Purpose:**

Working memory (WM) represents the brain’s ability to maintain information in a readily available state for short periods of time. This study examines the resting-state cortical activity patterns that are most associated with performance on a difficult working-memory task.

**Methods:**

Magnetoencephalographic (MEG) band-passed (delta/theta (1–7 Hz), alpha (8–13 Hz), beta (14–30 Hz)) and sensor based regional power was collected in a population of adult men (18–28 yrs, n = 24) in both an eyes-closed and eyes-open resting state. The normalized power within each resting state condition as well as the normalized change in power between eyes closed and open (zECO) were correlated with performance on a WM task. The regional and band-limited measures that were most associated with performance were then combined using singular value decomposition (SVD) to determine the degree to which zECO power was associated with performance on the three-back verbal WM task.

**Results:**

Changes in power from eyes closed to open revealed a significant decrease in power in all band-widths that was most pronounced in the posterior brain regions (delta/theta band). zECO right posterior frontal and parietal cortex delta/theta power were found to be inversely correlated with three-back working memory performance. The SVD evaluation of the most correlated zECO metrics then provided a singular measure that was highly correlated with three-back performance (r = −0.73, p<0.0001).

**Conclusion:**

Our results indicate that there is an association between WM performance and changes in resting-state power (right posterior frontal and parietal delta/theta power). Moreover, an SVD of the most associated zECO measures produces a composite resting-state metric of regional neural oscillatory power that has an improved association with WM performance. To our knowledge, this is the first investigation that has found that changes in resting state electromagnetic neural patterns are highly associated with verbal working memory performance.

## Introduction

Working memory (WM) consists of several interacting brain processes that are involved in both maintaining and manipulating information for short periods of time [1], [2].

The neural mechanisms contributing to WM have been a major focus of the neurosciences, with progress in this field being applied to investigations of a wide array of brain pathologies, education, learning, as well as furthering our basic understanding of the brain [2]–[8].

The ability to selectively attend to relevant information has been described as the capacity to focus our cognitive resources to our goals. Selective attention is thought to be a major facet of working memory that engages relevant sensory networks and inhibits irrelevant brain networks [9]–[12]. Some investigators suggest that selective attention and WM are supported by common neural mechanisms [2], [12], [13]. Investigations into these mechanisms have found that lapses in selective attention are associated with neurophysiological changes in cortical processing [13]. Both selective attention and physiological arousal, which has previously been described as an energetic state at a point in time, have been linked to the prefrontal and parietal cortices, suggesting that there may be a functional overlap between these two processes [12], [14]–[16]. Decreased levels of cortical arousal also appear to correlate with performance on cognitive tasks [15]. Thus, it appears that either task-related lapses in attention or decreased levels of cortical arousal can be detected physiologically and are linked to decreased cognitive performance [14], [15].

The importance of selective attention and arousal in working memory has been further strengthened by recent hemodynamic investigations showing associations between resting-state brain patterns and working memory performance [17]–[20]. Sala-Llonch et al. have hypothesized that resting-state brain activity may regulate top-down selective attention and arousal, which prepare brain networks for WM functions [19]. These studies suggest that markers of spontaneous neural activity during the resting state may allow for the measurement of selective attention and arousal networks, and thus predict WM proficiency.

Recent research demonstrates that magnetoencephalography can be used to detect correlated networks of brain activity during working memory [21]–[23]. The current study investigated resting-state cortical arousal and selective attention using magnetoencephalography (MEG). We hypothesized that individuals demonstrating higher levels of cortical arousal and higher levels of selective attention during a pre-task resting state would perform better on a subsequently performed WM task. As the optimal measures of both selective attention and arousal were not known, all regional measurements and band-limited (delta/theta, alpha, beta) cortical power measurements were used to determine if certain patterns of resting-state brain activity were associated with WM performance. Both the eyes-open and eyes-closed resting state were used, as it was also unclear which condition would be more associated with WM proficiency. Finally, we calculated the regional and band-limited changes in normalized power between the eyes closed and open conditions to identify individual differences in neural activity in the absence of visual stimulation. We hypothesized that a metric reflecting the changes in cortical activation and arousal occurring between eyes closed and open would provide additional information regarding each individual’s resting-state cortical function. These resting-state neurophysiological measures were then compared to WM performance to determine whether MEG-detected resting-state cortical activity was associated with WM performance.

## Materials and Methods

### Participants

The data were obtained from 24 adult male participants (1 left handed, Age: 18–28, Avg. = 22 yrs, Std = 3.0 yrs). All participants took part in this study after submitting signed informed consent. No participant had a history of psychiatric or neurologic conditions, trauma, or substance abuse. One subject was removed from the study as an outlier due to performance (three-back d′ >3 std dev. away from group d′ mean (see experimental protocol below)). Experimental procedures were approved by the University of California, San Diego (UCSD) Human Research Protections Program.

### Experimental Protocol

Before data collection, the study protocol was explained to each participant so that they were aware a working memory task would follow the resting state conditions, but they were not explicitly instructed to prepare for the task. To assess resting-state activity, participants were first asked to rest quietly with their eyes closed for four minutes and were then asked to fixate on a back-illuminated screen for another four minutes. This process was then repeated to obtain a total of 8 minutes in each resting-state condition. Both continuous video camera surveillance and real-time monitoring of MEG recordings for slow wave generation were used to ensure no participants fell asleep during the experimental protocol.

**Figure 1:**
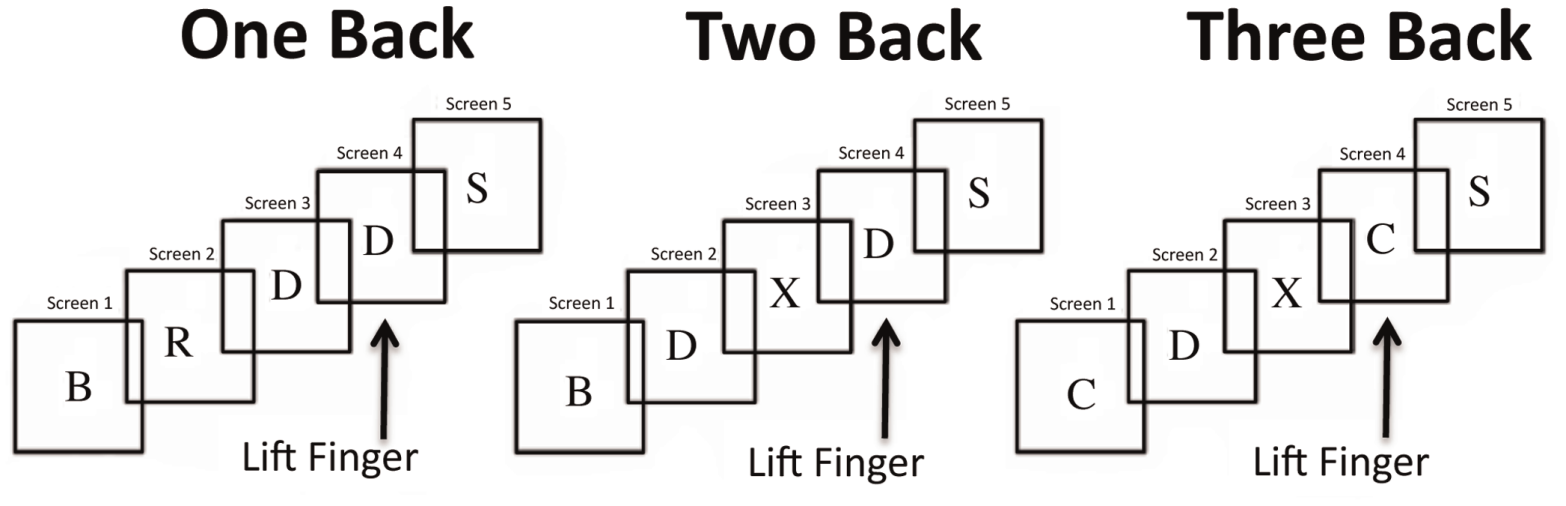
A diagram of the three n-back task conditions.

The subsequently performed verbal n-back WM task, adapted from Braver et al., was used to provide a metric of working memory performance [24]. Task stimuli consisted of letters presented on the same back projection screen as above using Presentation 14.5 (Neurobehavioral systems inc; Albany, CA). Participants performed two blocks of each of four conditions serially with a rest period between blocks. In the zero-back condition, included as a control because of minimal WM requirements, participants responded by raising the right index finger when a pre-specified letter appeared. In the one-back condition, participants indicated when the stimulus on the screen matched the previous letter by raising their right index finger. During the two-back condition participants were instructed to indicate when the presented stimulus matched the letter presented two before, and the three-back task similarly asked participants to respond when the stimulus matched the third previous letter (Figure 1). Responses were recorded using a non-metallic touchpad (Elekta-Neuromag, Helsinki, Finland). Behavioral response data was combined with stimuli presentation data on a single output channel. Matlab 10 b (Mathworks Inc, Natick, MA) was then used to perform automated behavioral analysis that was then verified by manual observation.

Each block consisted of 100 trials with each stimulus being presented for 500 ms and 1.5 seconds being allowed for response before the next stimulus. The duration between the onset of subsequent stimuli was 2 seconds regardless of participant responses. There was a short training session prior to each new condition. Each block of trials lasted roughly four minutes. Across conditions, true target stimuli accounted for 30% (3% std dev.) of all presented stimuli. Performance was evaluated using the d prime (d′) measure, which is the difference between the z-scores of the true positive rate and false positive rate. d′ = z(hit rate)- z(false alarm rate). Statistical analyses were calculated using Matlab 10 b as well as SPSS (v12.0, SPSS, Inc. Chicago, IL). Data can be obtained by contacting the corresponding author (Heister).

### MEG Data Acquisition

MEG activity was recorded continuously for the duration of the resting-state conditions by a VectorView whole-head MEG system (Elekta-Neuromag, Helsinki, Finland), using the 204 gradiometer channels in an enhanced multi-layer magnetically shielded room (IMEDCO-AG, Hägendorf, Switzerland) [25]. MEG data were acquired at 1000 Hz with a hardware band-pass filter at 0.1–330 Hz. Data were first visually inspected for large sources of noise and truncated to remove the first and last 10 seconds of data from each file. The signal space separation with temporal extension (SSST) package (MaxFilter) was applied to all MEG recordings to remove residual artifacts due to eye movements and/or cardiac signals [26], [27]. Independent component analysis was also performed to remove remaining eye-blink and cardiac signals. Precautions were taken to ensure head stability: foam wedges were inserted between the subject's head and the inside of the unit, and a Velcro strap was placed under the subject's chin and anchored in superior and posterior axes. The head positions were measured before each session and we ensured that the head movement across different sessions was less than 5 mm (usually 2–3 mm).

### Signal Processing

MEG data were collected in the eyes-open and eyes-closed conditions. Power analyses were conducted for the MEG data using Finite impulse response (FIR) filtering separated into bandwidths: delta/theta (1–7 Hz), alpha (8–13 Hz), and beta (14–30 Hz). All band-passed signals used edges consisting of 10% of each limit (e.g. 1–4 Hz used 0.1 and 0.4 Hz edges). Power amplitude was calculated by squaring the absolute value of the discrete time analytical signal obtained via the Hilbert transform of each band-passed signal. Power measurements for each channel were then averaged across each experimental block (roughly 4 minutes, as described above). Brain regions were separated into 10 cortical areas (i.e. left and right anterior-frontal, posterior-frontal, temporal, parietal, and occipital) based on sensor locations.

### Power Analysis and Performance Correlation

Regional power measurements in each (1–7 Hz, 8–13 Hz, 14–30 Hz) frequency band-passed signal were analyzed across the two conditions (eyes-open vs. eyes-closed). Before correlating these measurements with working memory performance signals were normalized by subtracting the mean and dividing by the standard deviation across all subjects in each band and region (z-score). To compare resting-state conditions, the eyes-open power was subtracted from the eyes-closed power in each region and band for each individual. Subsequently, each comparison was then z-score normalized across subjects. This metric was thus a z-score of the eyes-closed minus eyes-open power (zECO).

Initial attempts to separate the delta/theta band into two bands found that the power in smaller frequency ranges did not correlate as well with performance. The Pearson correlation coefficient was used to calculate which condition, region, and band-limited measures were most correlated with working-memory performance.

### Singular Value Decomposition

Makeig and Jung (1995) have previously established that electroencephalographic changes that relate to performance during an attention task can be isolated to a single principal component [28]. In a similar fashion, we hypothesized that our sensor based regional MEG recordings may reflect overlapping cortical processes and that performing a singular value decomposition (SVD) of our data had the advantage of finding the dominant pattern in this data and also to reduce the number of comparisons in our analysis. The SVD is a data-driven process that allows for the factorization of a data set matrix defined as X into orthogonal components and is calculated as follows:
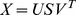



X is a data matrix with M rows and N columns that is then decomposed into a U matrix (M×N) that provides the best approximation of the column data in a lower rank for each mode. V^T^ is the transposed V matrix (N×N) that provides the best approximation of the row data in a lower rank for each mode. The diagonal components of the S matrix (N×N) are the singular value scaling factors for each mode and are arranged in descending order. SVD is similar to principal component analysis in that if each data column is normalized with a mean of zero, U*S of the SVD is equal to the principal component scores.

## Results

### Working Memory Performance

**Figure 2:**
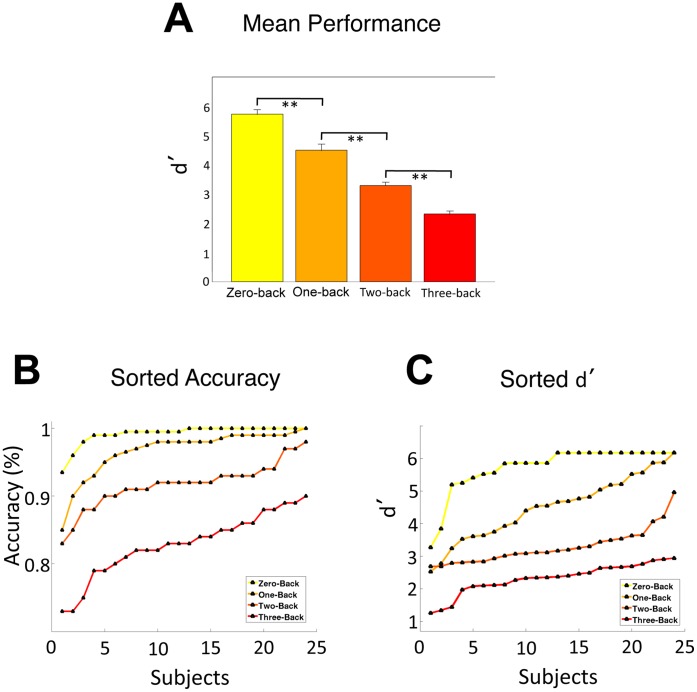
A: Mean Performance (± standard error). Performance significantly decreased with increasing task difficulty. B: Individual subject accuracy for each n-back condition. The three-back condition has a more linear distribution than the other two conditions and no ceiling effect (linear regression fit: zero-back r^2^ = 0.46; one-back r^2^ = 0.66; two-back r^2^ = 0.85; three-back r^2^ = 0.93). C: Individual subject d′ for each n-back condition. (linear regression fit: zero-back r^2^ = 0.58; one-back r^2^ = 0.98; two-back r^2^ = 0.81; three-back r^2^ = 0.89) ** = p<0.01, Bonferroni corrected.

Mean d′ progressively decreased as the n-back task became more difficult (zero-back: 5.7±0.7, one-back: 4.5±0.2, two-back: 3.3±0.1, three-back: 2.3±0.08; p<0.001 for all pairwise comparisons; Figure 2A). Individually sorted accuracy and d′ measures in each condition revealed that three-back performance was the most appropriate condition for stratifying individuals according to working-memory proficiency as it had no ceiling effect and displayed the most linear distribution of performances (accuracy linear regression fit: zero-back r^2^ = 0. 46; one-back r^2^ = 0.66; two-back r^2^ = 0.85; three-back r^2^ = 0.93; d′ linear regression fit: zero-back r^2^ = 0.58; one-back r^2^ = 0.98; two-back r^2^ = 0.81; three-back r^2^ = 0.89; Figure 2B, 2C). While both the three-back accuracy scores and d′ measures were found to have linear distributions, the three-back, d′ measure was used as a metric for working memory performance because it evaluated the ability of our subjects’ to identify correct stimuli without the influence of positive or negative response bias [29].

### Regional and Band Changes from Eyes Closed to Eyes Open

**Figure 3:**
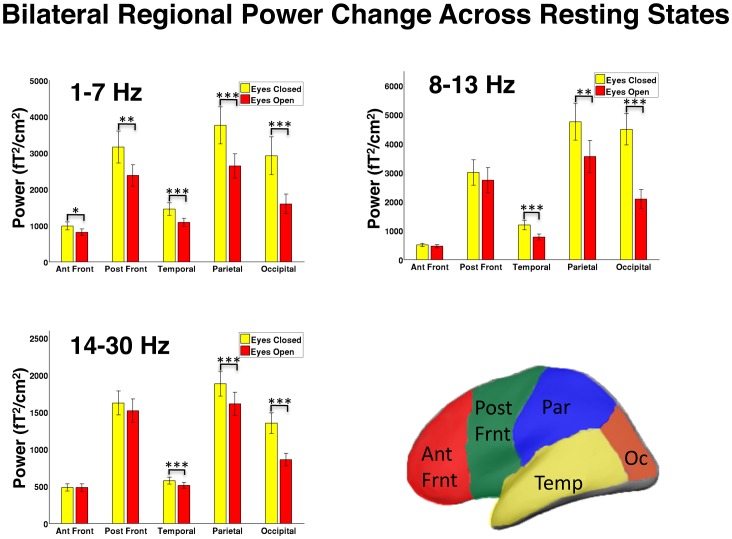
Regional and condition power analyses. Repeated measures ANOVA showing power amplitude decreases from eyes closed to eyes open. Delta/theta band changes between the conditions are particularly pronounced, and significant for all bands. Alpha power also had significant power decreases in the temporal lobes and posterior aspects of the brain. Similarly, beta group analysis showed significant power decreases in the temporal, parietal, and occipital lobes. A representative diagram is displayed of sensor-based separation of brain regions. aFr = anterior frontal cortex, pFr = posterior frontal cortex, Par = parietal cortex, Temp = temporal cortex, Oc = occipital cortex. * = p<0.05, ** = p<0.01, *** = p<0.001.

Further analyses were next conducted on condition, regional, and frequency band-limited power measurements. An initial inspection of the data found no significant difference between hemispheres in any frequency band (p>0.05). Thus, data were collapsed across hemispheres for these group analyses. A 2-factor repeated measures ANOVA was performed to test for changes in power across regions (anterior frontal, posterior frontal, temporal, parietal, occipital) and conditions (eyes open and eyes closed) for each frequency band. The Huynh-Feldt correction was used to adjust for heterogeneity of variance in multiple degrees-of-freedom tests. Figure 3 displays graphs of the results. In the delta/theta band, there was a significant interaction of region and condition effects (p<0.001). Power significantly decreased in all regions in the eyes open relative to the closed condition (anterior frontal: −173 fT^2^/cm^2^, p<0.05; posterior frontal: −786 fT^2^/cm^2^, p<0.01; temporal: −364 fT^2^/cm^2^, p<0.001; parietal: −1119 fT^2^/cm^2^, p<0.001; −1326 occipital fT^2^/cm^2^, p<0.001). In the alpha band, there was a significant interaction of region and condition effects (p<0.001). Power was also significantly decreased in the eyes open relative to the eyes closed condition (temporal −417 fT^2^/cm^2^, p<0.001; parietal −1201 fT^2^/cm^2^, p<0.01; occipital −2405 fT^2^/cm^2^, p<0.001). The beta band also exhibited a significant interaction between condition and region (p<0.001), with regional decreases in the eyes open relative to closed condition (temporal −63 fT^2^/cm^2^, p<0.001; parietal −271 fT^2^/cm^2^, p<0.001; occipital −492 fT^2^/cm^2^, p<0.001).

### Correlations of Cortical Power and WM Performance

**Figure 4:**
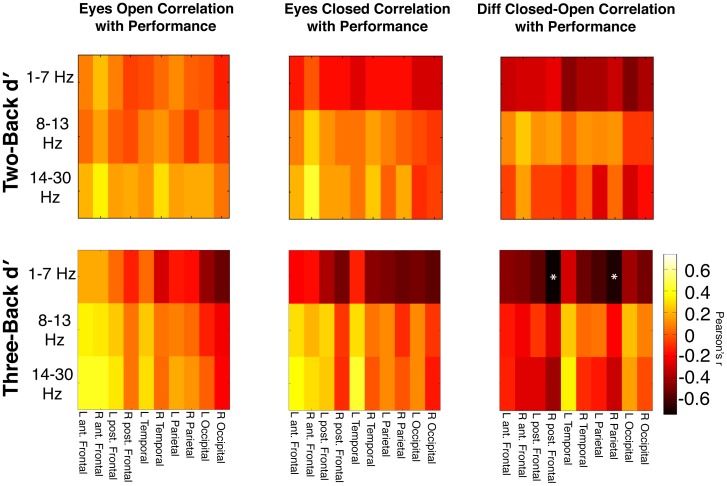
Pearson correlation coefficient (r) values between both two-back and three-back performance and normalized power in each region and band. The normalized difference between eyes closed and open power (zECO) revealed negative associations with two-back performance and left temporal and occipital power. Three-back performance was found to be inversely associated with delta/theta eyes open power in both the left and right occipital regions (1–7 Hz, R/L occipital). Eyes closed bilateral frontal and left temporal alpha and beta power were mildly positively associated with three-back working memory performance. The delta/theta band power in the bilateral occipital, bilateral parietal, right posterior frontal, and right temporal regions was most associated with three-back WM performance. The zECO power revealed negative associations with three-back performance in several delta/theta regions and significant correlations in the right posterior frontal and right parietal regions. * = Bonferroni corrected p<0.05.

Figure 4 displays the Pearson correlation r value for each band-limited comparison of neuronal activity and WM performance in all resting conditions as compared to both two-back and three-back d′ performance. While there were several interesting associations as described below, no individual comparison in either the eyes-open or eyes-closed conditions survived Bonferroni multiple comparisons correction for significance. The two-back comparisons did not show any significant correlations but are included to allow for comparison with three-back data. In the three-back eyes-open comparison, performance was inversely associated with delta/theta power in both the left and right occipital regions. In the three-back eyes-closed comparison, alpha and beta power in the bilateral frontal and left temporal regions was mildly positively associated with working memory performance. Additionally, several regions in the delta/theta band exhibited inverse associations with three-back WM performance.

Next we correlated WM performance with the normalized difference in power between the eyes closed and open conditions (zECO). Figure 4 found that delta/theta zECO power in multiple regions had a mild inverse non-significant association with two-back performance. Figure 4 demonstrates that increases in delta/theta zECO power in many regions negatively correlated with WM performance, but the right posterior frontal and parietal regions were found to have significant correlations that survived Bonferroni correction(r = −0.72, p<0.05 with Bonferroni correction; r = −0.696, p<0.05 with Bonferroni correction; df = 22, respectively). Thus, individuals with better three-back WM performance showed little change or decreases in right posterior frontal and parietal delta/theta zECO power.

### Selection of Condition

**Figure 5:**
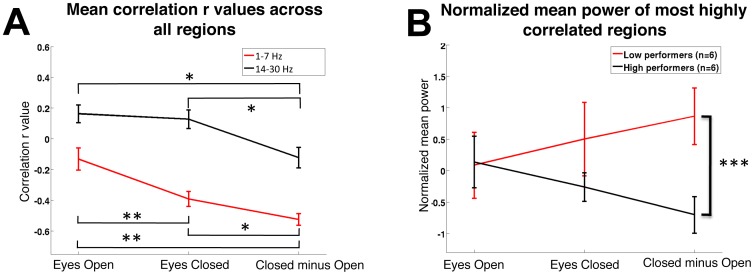
A: *r* values for all regions across conditions show a larger inverse correlation and a decrease in positive correlation in the zECO conditions. B: Using the most highly correlated normalized band and region comparisons (delta/theta right posterior frontal, delta/theta right parietal, beta right posterior frontal), high performers and low performers exhibit large differences in the zECO power across resting-state conditions. * = Bonferroni corrected p<0.05. ** = Bonferroni corrected p<0.01. *** = single comparison p<0.001.

We next aimed to quantitatively establish which resting state condition measurements were most associated with working memory performance. Due to a lack of significant two-back comparisons, only the three back comparisons were further analyzed. The *r* values of all regions in the three-back delta/theta and beta bands were combined within each condition. The alpha band was excluded from further analysis due to the lack of a discernible power/performance trend (Figure 4). This analysis found that the zECO *r* values had significantly larger negative correlations than either eyes open or closed conditions alone (Fisher *z* corrected; 1–7 Hz, Open vs. zECO Bonferroni corrected p<0.01, Open vs. Closed Bonferroni corrected p<0.01, Closed vs. zECO Bonferroni corrected p<0.05; 14–30 Hz, Open vs. zECO Bonferroni corrected p<0.05, Closed vs. zECO Bonferroni corrected p<0.05; Figure 5A).

### Selection of Regions most Correlated with Performance

Next we sought to identify the regions and frequency-limited comparisons that most correlated with three-back WM performance. In order to identify the region and band measures that were most highly correlated with performance, the absolute value of region and band correlation *r* values were converted to band-limited z-scores. Regions in the delta/theta band and beta band that were more than 1 standard deviation from the remainder of the band-limited comparisons were selected. These criteria isolated the right posterior frontal and right parietal regions in the delta/theta band, and the right posterior frontal region in the beta band.

### Performance Classifications

In order to further evaluate changes in power and working memory performance, participants were then separated into WM three-back performance-based groups: high performers (highest 25% of population; d′≥2.67 n = 6), and low performers (lowest 25% of population; d′≤2.1 n = 6). The normalized mean power of the regions and bands that were most associated with performance as described above were then compared between these two performance groups to show the differences in cortical activity in these regions between groups. Low performers and high performers were observed to have similar eyes-open power in these selected regions, but low performers had increased power during the eyes-closed state, while high performers exhibited decreases in power in the eyes-closed state. This resulted in zECO values that were much higher in low performers than high performers (p<0.001**) (** Figure 5B, 6A demonstrates selected regions that were most highly associated with WM performance).

**Figure 6:**
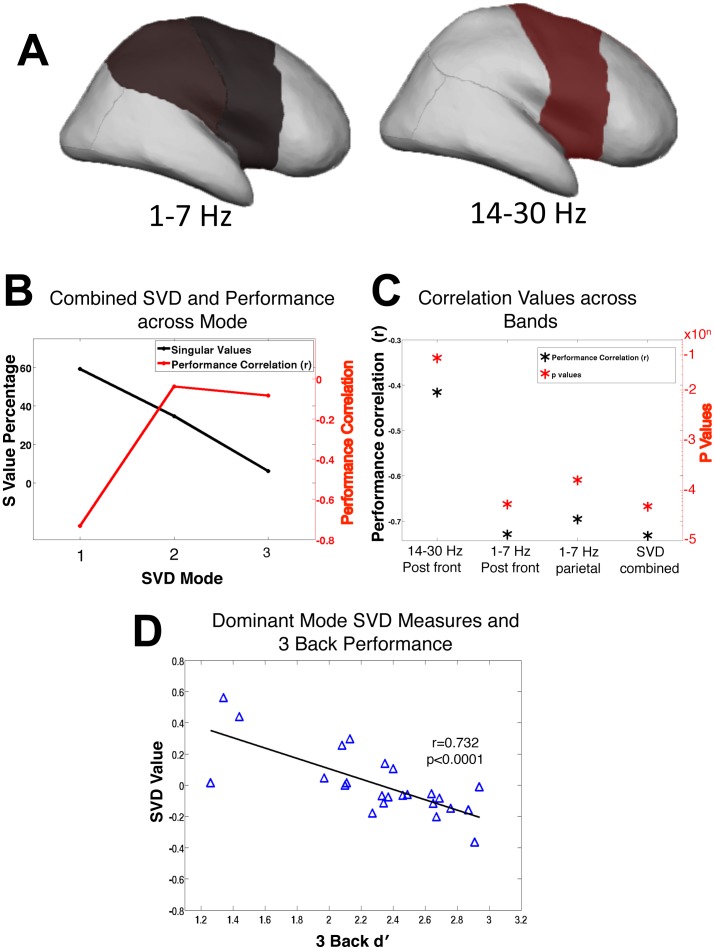
SVD combined regions and association with working memory performance. A: Representative diagram of the delta/theta and beta regions used in the SVD (delta/theta right posterior frontal, delta/theta right parietal, beta right posterior frontal regions). B: The singular values for the SVD matrix indicated that 59% of the variability in the data was present in the first mode, and that this mode correlated with WM performance. The second and third modes contained 35% and 6% of the variability respectively, but these modes did not correlate with performance. C: Displays individual Pearson’s correlation values (r) and significance values (p) for selected region comparisons as well as the calculated dominant (first) mode SVD r and p values. D: Scatter plot showing a strong association between the SVD values of the first mode and three-back d′scores.

### SVD Analysis

We found that the comparisons most associated with performance were from neighboring brain regions. As such, these measures contained overlapping information that can be more clearly demonstrated using SVD, which isolates the dominant patterns in the data and produces an individual resting-state metric. This metric was more highly associated with WM performance than any individual comparison. We first used the selection criteria described above to identify the region and frequency zECO power measurements that most correlated with performance (Figure 6A).

An SVD was then performed using the normalized power across subjects from these three comparisons (1–7 Hz right posterior frontal and parietal, 14–30 Hz right posterior frontal). This compiled data formed a 24×3 data matrix (24 subjects × 3 selected band/region comparisons) that was then decomposed into a 24×3 U matrix (subjects × modes), a 3×3 singular value matrix, and a 3×3 transposed V matrix (selected band/region comparisons × modes).

Evaluation of the singular (S) value for each mode revealed that 59% of the variance in the data was present in the first mode, whereas only 35% and 6% of the variance were in the second and third modes, respectively. Correlating the weighting arrays from each mode revealed that the first mode highly correlated with WM performance (r = −0.732) in contrast to the second (r = −0.038) and the third modes (r = −0.084). Thus, the performance-related variance in the data appears to be restricted to the first mode (Figure 6B). The resulting first mode of the U matrix was found to be mildly more correlated with the three-back d′ measure than the single most correlated comparison (right posterior frontal r = −0.72 versus the SVD first mode r = −0.73, p<0.0001, df = 22, Figure 6C, 6D). The negative correlation of the SVD values and WM d′ indicates that lower-performing subjects had larger decreases in power from eyes closed to open in the right posterior frontal and parietal regions whereas higher-performing subjects tended to show little change or increases in power from eyes closed to open in the posterior frontal and parietal regions (Figure 6D). To establish that this effect is not a result of the three lowest performers, this analysis was then repeated with these three subjects removed and the Pearson correlation remained significant (r = −0.63, p<0.005, n = 21).

## Discussion

Resting-state measures can be useful for understanding individual differences in the variability of resting-state cortical activity and perhaps the extent to which resting-state cortical activity contributes to cognitive functioning, including WM. To our knowledge, this is the first study to show that individual changes in delta/theta power (1–7 Hz; right posterior frontal and parietal regions) from eyes closed to eyes open are highly associated with performance on a subsequent three-back WM task. Furthermore, changes in power from eyes closed to open in select regions can be combined into the most dominant (i.e. first) mode of an SVD to provide a singular resting-state measure that is more highly associated with WM performance than any individual measure.

An association of resting-state cortical activity and WM performance has previously been observed using hemodynamic and neurophysiological markers [17]–[19], [30]–[32]. More specifically, some investigators reported that resting-state activation in the parietal region is consistently linked to an attention network and n-back performance [19], [33]–[35]. Activation in the right frontal and parietal regions have previously been linked to the engagement of visuospatial processing and selective attention [19], [33]–[39]. Additionally, neurophysiological measures of selective attention and cortical arousal (e.g. larger delta/theta and beta power) are associated with WM performance and WM capacity [2], [13], [15], [39]–[43].

Our results demonstrated that individuals with poorer three-back WM showed larger increases in right posterior frontal and parietal delta/theta from eyes closed to open conditions. In contrast, higher performing subjects tended to have less change or a decrease in power in these regions across resting-state conditions (Figure 5B). These findings are consistent with prior investigations suggesting that patterns of resting-state brain activity in the right posterior frontal and parietal regions may be markers for the engagement of selective attention or cortical arousal. While selective attention has classically been considered to be focused on external perceptual inputs, it has recently been proposed that this entity can also be directed toward internal processes as well [9], [10]. We suggest that lower cortical arousal was associated with both higher eyes closed delta/theta power in our selected regions and also associated with lower WM performance. We speculate that the variability of the normalized change in power across resting states may result from the engagement of internal selective attention or increased cortical arousal occurring in high WM performers in the eyes open fixation task.

As there were several neighboring brain regions that correlated with performance, we found that the association between resting-state cortical processes and WM performance was strengthened by using the dominant pattern in the data across multiple regions via SVD. While the SVD did not provide dramatic improvements in the power associations, it proved useful for combining several resting-state measurements into a single measure that was more highly associated with performance than any one regional or band limited measure. SVD is a data driven method that searches for the dominant patterns in the data while, isolating unrelated variation to less dominant modes, and minimizes the problem of multiple comparisons.

Previous groups have found that tonic alpha power, phasic alpha power, and peak alpha frequency correlated with working memory processing and performance, while the current investigation found very little correlation between performance and resting state alpha power [41], [44]–[45]. This discrepancy is likely the result of previous investigations using power recorded during the task while this study was interested in resting state power and behavior correlations. Furthermore, differences in methodology (i.e. electroencephalography versus magnetoencephalography based, different cognitive measures of working memory, and different population demographics) make direct comparisons of our data with prior investigations difficult [46].

The results of this investigation were also inconsistent with a recent EEG study that found a small but significant positive correlation between an eyes-closed resting state relative theta power, and performance on an attention and recall task [47]. We suggest that this may in large part be due to our use of MEG data from young adult males (mean 22 years of age) versus the use of EEG data from an elderly population (mean = 61 years of age) comprising more than 60% females. These results suggest that while certain resting-state measures may be associated with performance, the regional and band-limited measures and level of association of these measures are likely dependent on population demographics and working memory task employed.

Initial attempts to separate the delta/theta band into two separate bands revealed that some participants produced this behavioral correlation in the lower (1–4 Hz) band whereas other subjects showed this correlation in the higher (4–7 Hz) band. These initial analyses failed to show that lower and higher bands differentiated the low and high WM performers. Moreover, these groups could not be separated on the basis of demographic characteristics or performance metrics. Thus, our data indicated that the physiological effect we were measuring extended across both bands in our population and a compiled 1–7 Hz band was the optimal measure for our purposes.

Previous studies have found measures of both cortical arousal and selective attention affect cognitive performance, and our findings that resting-state power measures are associated with WM performance is consistent with these prior investigations, although direct measurements of arousal or selective attention were not collected in this study [42], [48]–[50]. Prior investigations into the role of the eyes open versus closed states have suggested that changes in alpha power were related to cortical arousal and changes in delta, theta, and beta power were more related to cortical activation [49]. This would suggest that it is the differential cortical activation of various resting state brain networks and not cortical arousal that has functional implications on cognitive task performance. More studies will be necessary to clarify the neural mechanisms leading to the strong correlation of WM task performance and changes in right posterior frontal and parietal power from eyes closed to open.

## References

[1] Goldman-Rakic PS (1995) Cellular Basis of Working Memory Review. Cell 14: 477–485. 10.1016/0896-6273(95)90304-6 7695894

[2] Baddeley A (2012) Working memory: theories, models, and controversies. Annual review of psychology 63: 1–29. 10.1146/annurev-psych-120710-100422 21961947

[3] Levy F, Farrow M (2001) Working memory in ADHD: prefrontal/parietal connections. Current drug targets 2: 347–352. 11732636 10.2174/1389450013348155

[4] O’Hearn K, Asato M, Ordaz S, Luna B (2008) Neurodevelopment and executive function in autism. Development and psychopathology 20: 1103–1132. 18838033 10.1017/S0954579408000527

[5] Conway ARa, Kane MJ, Engle RW (2003) Working memory capacity and its relation to general intelligence. Trends in Cognitive Sciences 7: 547–552. 14643371 10.1016/j.tics.2003.10.005

[6] Martinussen R, Hayden J, Hogg-Johnson S, Tannock R (2005) A meta-analysis of working memory impairments in children with attention-deficit/hyperactivity disorder. Journal of the American Academy of Child and Adolescent Psychiatry 44: 377–384. 15782085 10.1097/01.chi.0000153228.72591.73

[7] McAllister TW, Flashman L a, Sparling MB, Saykin AJ (2004) Working memory deficits after traumatic brain injury: catecholaminergic mechanisms and prospects for treatment – a review. Brain injury: [BI] 18: 331–350. 10.1080/02699050310001617370 14742148

[8] Vogels TP, Rajan K, Abbott LF (2005) Neural network dynamics. Annual review of neuroscience 28: 357–376. 10.1146/annurev.neuro.28.061604.135637 16022600

[9] Chun MM (2011) Visual working memory as visual attention sustained internally over time. Neuropsychologia 49: 1407–1409. 21295047 10.1016/j.neuropsychologia.2011.01.029

[10] Chun MM, Golomb JD, Turk-Browne NB (2011) A taxonomy of external and internal attention. Annual review of psychology 62: 73–101. 10.1146/annurev.psych.093008.100427 19575619

[11] Wang X (2010) Neurophysiological and Computational Principles of Cortical Rhythms in Cognition. Physiological Reviews: 1195–1268. 10.1152/physrev.00035.2008 PMC2923921 20664082

[12] Gazzaley A, Nobre AC (2012) Top-down modulation: bridging selective attention and working memory. Trends in cognitive sciences 16: 129–135. 22209601 10.1016/j.tics.2011.11.014 PMC3510782

[13] Rutman AM, Clapp WC, Chadick JZ, Gazzaley A (2010) memory performance. Neurology 22: 1224–1234. 10.1162/jocn.2009.21257 PMC2842470 19413473

[14] Makeig S, Jung TP, Sejnowski TJ (2000) Awareness during drowsiness: dynamics and electrophysiological correlates. Canadian journal of experimental psychology = Revue canadienne de psychologie expérimentale 54: 266–273. 11195717 10.1037/h0087346

[15] Makeig S, Jung TP (1996) Tonic, phasic, and transient EEG correlates of auditory awareness in drowsiness. Brain research Cognitive brain research 4: 15–25. 8813409 10.1016/0926-6410(95)00042-9

[16] Barry R, Clarke A, McCarthy R, Selikowitz M, Rushby J (2005) Arousal and Activation in a Continuous Performance Task: An Exploration of State Effects in Normal Children. Journal of Psychophysiology 19 2: 91–99.

[17] Hampson M, Driesen NR, Skudlarski P, Gore JC, Constable RT (2006) Brain connectivity related to working memory performance. The Journal of neuroscience: the official journal of the Society for Neuroscience 26: 13338–13343. 17182784 10.1523/JNEUROSCI.3408-06.2006 PMC2677699

[18] Hampson M, Driesen N, Roth JK, Gore JC, Todd R (2010) Functional connectivity between task-positive and task-negative brain areas and its relation to working memory performance. Magnetic resonance imaging 28: 1051–1057. 20409665 10.1016/j.mri.2010.03.021 PMC2936669

[19] Sala-Llonch R, Peña-Gómez C, Arenaza-Urquijo EM, Vidal-Piñeiro D, Bargalló N, et al.. (2011) Brain connectivity during resting state and subsequent working memory task predicts behavioural performance. Cortex: 1–10. 10.1016/j.cortex.2011.07.006 21872853

[20] Esposito F, Aragri a, Latorre V, Popolizio T, Scarabino T, et al (2009) Does the default-mode functional connectivity of the brain correlate with working-memory performances? Archives italiennes de biologie 147: 11–20. 19678593

[21] Brookes MJ, Wood JR, Stevenson CM, Zumer JM, White TP, et al (2011) Changes in brain network activity during working memory tasks: a magnetoencephalography study. NeuroImage 55: 1804–1815. 21044687 10.1016/j.neuroimage.2010.10.074 PMC6485426

[22] Jensen O, Gelfand J, Kounios J, Lisman JE (2002) Oscillations in the alpha band (9–12 Hz) increase with memory load during retention in a short-term memory task. Cerebral cortex (New York, NY: 1991) 12: 877–882. 10.1093/cercor/12.8.877 12122036

[23] Jensen O, Tesche CD (2002) SHORT COMMUNICATION Frontal theta activity in humans increases with memory load in a working memory task. Neuroscience 15: 1395–1399. 10.1046/j.1460-9568.2002.01975.x 11994134

[24] Braver TS, Cohen JD, Nystrom LE, Jonides J, Smith EE, et al (1997) A parametric study of prefrontal cortex involvement in human working memory. NeuroImage 5: 49–62. 9038284 10.1006/nimg.1996.0247

[25] Cohen D, Schläpfer U, Ahlfors S, Hämäläinen M, Halgren E (2002) New Six-Layer Magnetically-Shielded Room for MEG. In: Nowak HHJ, Gießler F, editors. Proceedings of the 13th International Conference on Biomagnetism. Jena, Germany: VDE Verlag. 919–921.

[26] Taulu S, Kajola M, Simola J (2004) Suppression of interference and artifacts by the Signal Space Separation Method. Brain topography 16: 269–275. 15379226 10.1023/b:brat.0000032864.93890.f9

[27] Song T, Gaa K, Cui L, Feffer L, Lee RR, et al (2008) Evaluation of signal space separation via simulation. Medical & biological engineering & computing 46: 923–932. 18196307 10.1007/s11517-007-0290-y

[28] Makeig S, Jung T (1995) Changes in alertness are a principal component of variance in the EEG spectrum. Neuroreport 7: 213–216. 8742454

[29] Macmillan NA, Creelman CD (2005) Detection Theory: A User’s Guide. 2nd ed. Mahwah, NJ: Lawrence Erlbaum Associates, Inc.

[30] Del Río D, Cuesta P, Bajo R, García-Pacios J, López-Higes R, et al (2012) Efficiency at rest: Magnetoencephalographic resting-state connectivity and individual differences in verbal working memory. International journal of psychophysiology: official journal of the International Organization of Psychophysiology 86: 160–167. 22940641 10.1016/j.ijpsycho.2012.08.011

[31] Zou Q, Ross TJ, Gu H, Geng X, Zuo X-N, et al.. (2012) Intrinsic resting-state activity predicts working memory brain activation and behavioral performance. Human brain mapping. In press. 10.1002/hbm.22136 PMC6870161 22711376

[32] Pyka M, Hahn T, Heider D, Krug A, Sommer J, et al.. (2012) Baseline activity predicts working memory load of preceding task condition. Human brain mapping. In press. 10.1002/hbm.22121 PMC6869906 22696432

[33] Périn B, Godefroy O, Fall S, De Marco G (2010) Alertness in young healthy subjects: an fMRI study of brain region interactivity enhanced by a warning signal. Brain and cognition 72: 271–281. 19875216 10.1016/j.bandc.2009.09.010

[34] Cavanna AE, Trimble MR (2006) The precuneus: a review of its functional anatomy and behavioural correlates. Brain: a journal of neurology 129: 564–583. 16399806 10.1093/brain/awl004

[35] Petersen SE, Posner MI (2012) The Attention System of the Human Brain: 20 Years After. Annual review of neuroscience. 10.1146/annurev-neuro-062111-150525 PMC3413263 22524787

[36] Sturm W, Willmes K (2001) On the functional neuroanatomy of intrinsic and phasic alertness. NeuroImage 14: S76–84. 11373136 10.1006/nimg.2001.0839

[37] Sturm W, Schmenk B, Fimm B, Specht K, Weis S, et al (2006) Spatial attention: more than intrinsic alerting? Experimental brain research Experimentelle Hirnforschung Expérimentation cérébrale 171: 16–25. 16307253 10.1007/s00221-005-0253-1

[38] Arruda JE, Walker Ka, Weiler MD, Valentino Da (1999) Validation of a right hemisphere vigilance system as measured by principal component and factor analyzed quantitative electroencephalogram. International journal of psychophysiology: official journal of the International Organization of Psychophysiology 32: 119–128. 10380946 10.1016/s0167-8760(99)00006-9

[39] Arruda JE, Zhang H, Amoss RT, Coburn KL, Aue WR (2009) Rhythmic oscillations in quantitative EEG measured during a continuous performance task. Applied psychophysiology and biofeedback 34: 7–16. 19057991 10.1007/s10484-008-9071-0

[40] Foucher JR, Otzenberger H, Gounot D (2004) Where arousal meets attention: a simultaneous fMRI and EEG recording study. NeuroImage 22: 688–697. 15193597 10.1016/j.neuroimage.2004.01.048

[41] Downar J, Crawley aP, Mikulis DJ, Davis KD (2000) A multimodal cortical network for the detection of changes in the sensory environment. Nature neuroscience 3: 277–283. 10700261 10.1038/72991

[42] VaezMousavi SM, Barry RJ, Clarke AR (2009) Individual differences in task-related activation and performance. Physiology & behavior 98: 326–330. 19545581 10.1016/j.physbeh.2009.06.007

[43] Klimesch W (1999) EEG alpha and theta oscillations reflect cognitive and memory performance: a review and analysis. Brain research Brain research reviews 29: 169–195. 10209231 10.1016/s0165-0173(98)00056-3

[44] Vogel EK, Machizawa MG (2004) Neural activity predicts individual differences in visual working memory capacity. Nature 428: 748–751. 15085132 10.1038/nature02447

[45] Richard Clark C, Veltmeyer MD, Hamilton RJ, Simms E, Paul R, et al (2004) Spontaneous alpha peak frequency predicts working memory performance across the age span. International journal of psychophysiology: official journal of the International Organization of Psychophysiology 53: 1–9. 15172130 10.1016/j.ijpsycho.2003.12.011

[46] Klimesch W, Vogt F, Doppelmayr M (1999) Interindividual differences in alpha and theta power reflect memory performance. Intelligence 27: 347–362.

[47] Vogt F, Doppelmayr M (2000) Interindividual Differences in Alpha and Theta Power Reflect Memory Performance. 27: 347–362.

[48] Finnigan S, Robertson IH (2011) Resting EEG theta power correlates with cognitive performance in healthy older adults. Psychophysiology 48: 1083–1087. 21729101 10.1111/j.1469-8986.2010.01173.x

[49] Barry RJ, Clarke AR, Johnstone SJ, Magee Ca, Rushby Ja (2007) EEG differences between eyes-closed and eyes-open resting conditions. Clinical neurophysiology: official journal of the International Federation of Clinical Neurophysiology 118: 2765–2773. 17911042 10.1016/j.clinph.2007.07.028

[50] Barry R (2004) EEG differences in children as a function of resting-state arousal level. Clinical Neurophysiology 115: 402–408. 14744583 10.1016/s1388-2457(03)00343-2

